# Smoldering myocarditis following immune checkpoint blockade

**DOI:** 10.1186/s40425-017-0296-4

**Published:** 2017-11-21

**Authors:** Timothy G. Norwood, Brian C. Westbrook, Douglas B. Johnson, Silvio H. Litovsky, Nina L. Terry, Svetlana B. McKee, Alan S. Gertler, Javid J. Moslehi, Robert M. Conry

**Affiliations:** 1 UAB School of Medicine, 1670 University Blvd, Birmingham, AL 35233 USA; 2 Vanderbilt University Medical Center, 1211 Medical Center Dr, Nashville, TN 37232 USA; 3 UAB School of Medicine, 1802 6th Ave S, Birmingham, AL 35233 USA; 4 UAB Medicine, 625 19th St S, Birmingham, AL 35233 USA; 5 UAB Medicine, 2145 Bonner Way, Birmingham, AL 35243 USA; 6 Vanderbilt School of Medicine, 220 Pierce Ave, Nashville, TN 37232 USA

**Keywords:** Myocarditis, Cardiotoxicity, Immune checkpoint blockade, Nivolumab, Ipilimumab, Melanoma, Troponin, Antibody, Cardio-oncology

## Abstract

**Background:**

Severe myocarditis associated with electrical conduction abnormalities and occasionally heart failure has been well documented following treatment with immune checkpoint blockade with an estimated incidence of less than 1%. However, the incidence, early detection, and management of less severe immune-related myocarditis are unknown since most immunotherapy trials have not included routine cardiac monitoring. Herein, we provide the first description of subclinical or smoldering myocarditis with minimal signs and symptoms following immune checkpoint blockade with a single dose of ipilimumab and nivolumab.

**Case presentation:**

Our patient was diagnosed with immune checkpoint blockade-induced myocarditis based upon an acute rise in serum cardiac troponin I beginning 2 weeks after the initial dose of ipilimumab/nivolumab consistent with the reported median onset of clinical myocarditis at 17 days, as well as a lack of other causes despite extensive cardiac evaluation. The patient initially presented with intractable nausea with no known gastrointestinal etiology. High dose glucocorticoid therapy led to rapid resolution of nausea and a four-fold decrease in troponin I over 4 days. Serum troponin I spiked again following a steroid taper to 13 times the upper limit of normal with endomyocardial biopsy revealing collagen fibrosis and lymphocytic inflammation predominantly comprised of CD8+ T cells consistent with chronic smoldering myocarditis. Serum anti-striated muscle antibodies were also detected with no evidence of rhabdomyolysis. Serum cardiac troponin I levels as an indicator of ongoing myocyte damage gradually improved with chronic prednisone at 10 mg daily. Late addition of intravenous immunoglobulin was associated with rapid normalization of creatine kinase-myocardial band.

**Conclusions:**

This case demonstrates that subclinical, smoldering myocarditis may occur following immune checkpoint blockade, with evidence of both humoral and cell-mediated immunity responsive to corticosteroid therapy. This experience supports early monitoring for myocarditis with serial electrocardiograms and serum troponin I determinations in large, prospective cohorts of patients receiving combination immune checkpoint blockade as early detection and initiation of immunosuppression may forestall fulminant presentation of this disease and limit myocardial damage.

## Background

Immune checkpoint blockade (CPB) with monoclonal antibodies such as ipilimumab targeting cytotoxic T-lymphocyte-associated protein 4 (CTLA-4) or nivolumab/pembrolizumab targeting programmed cell death protein 1 (PD-1) have revolutionized the treatment of advanced melanoma and many other common malignancies. These agents activate T cells by inhibiting the interaction of T cell surface proteins (CTLA-4/PD-1) with their respective ligands on antigen presenting cells and tumor cells. Under normal conditions, these interactions limit autoimmune T cell activation against host tissues and induce exhaustion of activated T cells to limit collateral damage at sites of infection. Blockade of these checkpoints unleashes antitumor immunity, but also facilitates autoreactivity against normal tissues which manifests clinically as immune-related adverse events (irAEs). Approximately 20% of patients receiving anti-PD1 therapy, 30% receiving ipilimumab monotherapy, and 55% receiving ipilimumab and nivolumab (ipi/nivo) combination therapy will experience serious irAEs including dermatitis, colitis, hypophysitis, hepatitis, pneumonitis, nephritis and, more rarely, myocarditis [1].

Severe myocarditis associated with chest pain, dyspnea, and electrical conduction delays has been well documented and is more frequent following ipi/nivo combination therapy, with an estimated incidence of 0.3% and 5 deaths among 8 cases from one database [2, 3]. A 2016 review of cardiotoxicity associated with CPB in this journal described four cases of acute myocarditis following ipilimumab with or without anti-PD1 therapy with two fatalities [4]. However, the incidence, early detection, and management of less severe immune-related myocarditis are unknown since most immunotherapy trials have not included routine cardiac monitoring using electrocardiogram (ECG) or serum cardiac troponin I (cTnI) measurements. Herein, we provide the first description of subclinical or “smoldering” myocarditis with minimal signs and symptoms following immune CPB with a single dose of ipi/nivo. Further, we suggest that early detection and initiation of corticosteroids may forestall fulminant presentation of this disease and limit myocardial damage.

## Methods

Clinical and laboratory data were collected by extraction of the electronic medical record. Weekly troponin I measurements were obtained in this patient treated with ipilimumab and nivolumab given prior reports of myocarditis. Serial cTnI measurements were made using the Siemens Centaur XP Immunoassay System at the University of Alabama at Birmingham Hospital Lab. Selected samples were also assayed using the Beckman Coulter UniCel DXI 800 Immunoassay System and concordance was verified. To exclude heterophilic antibodies as a cause for false-positivity, selected samples were assayed on both the Siemens and Beckman Coulter instruments with and without polyethylene glycol pre-incubation shown to remove 98% of heterophilic antibody interference [5].

## Case presentation

A 49-year-old Caucasian female with no cardiac risk factors aside from mild hyperlipidemia underwent wide local excision and regional lymphadenectomy to remove a BRAF wild type cutaneous melanoma of the right anterior chest involving a single right axillary sentinel lymph node (Stage IIIA). She received 1 month of adjuvant intravenous interferon and remained clinically disease-free for 8 years before biopsy confirmation of multiple, bilateral pulmonary metastases. As initial therapy for metastatic melanoma, she received ipilimumab at 3 mg/kg and nivolumab at 1 mg/kg. Serum cTnI levels were within the normal range (0.02–0.06 ng/mL) 2 weeks prior to treatment and again on day 1 of ipi/nivo. Two days after initiating treatment, she experienced painless anterior neck swelling representing treatment-related thyroiditis which resolved spontaneously but subsequently required thyroid hormone supplementation.

**Fig. 1:**
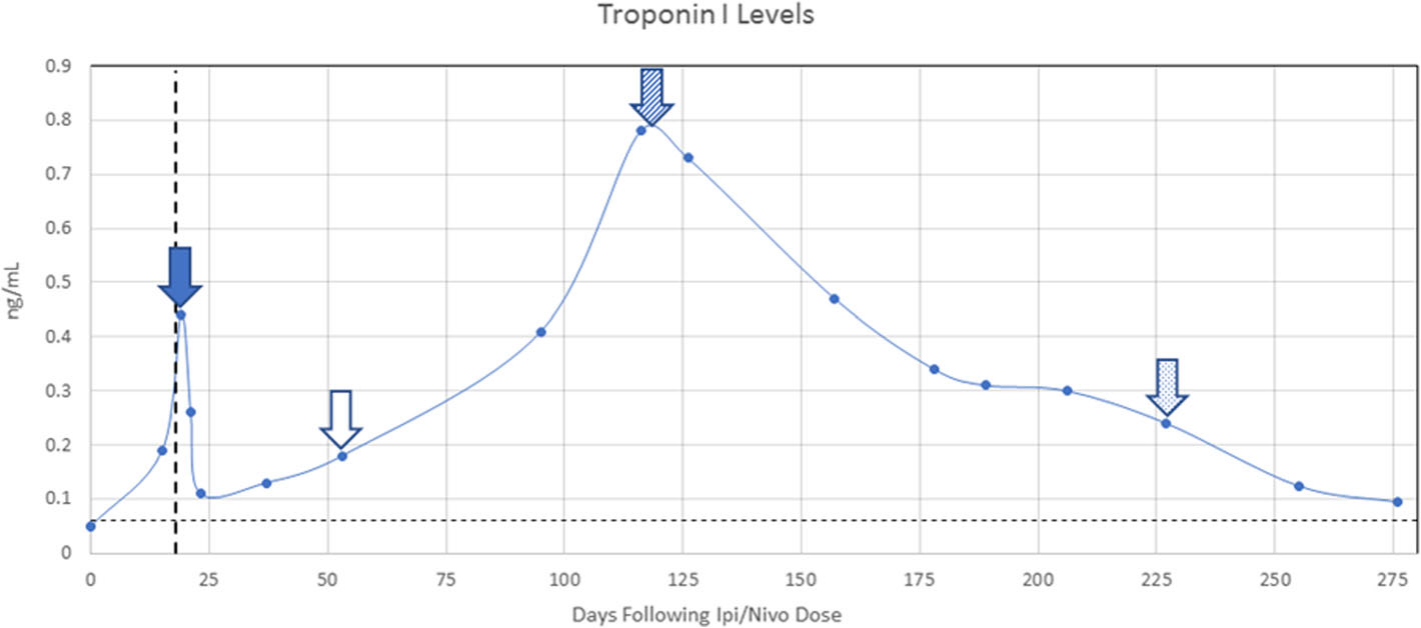
Serial Cardiac Troponin I levels following a single dose of Ipi/Nivo. High dose glucocorticoids started , end of steroid taper , initiation of prednisone at 10 mg daily,  and initiation of intravenous immunoglobulin . Vertical dashed line represents the previously reported median onset of clinical myocarditis at 17 days following immune CPB. Horizontal dashed line represents the upper limit of normal for serum cTnI at 0.06 ng/mL

**Fig. 2:**
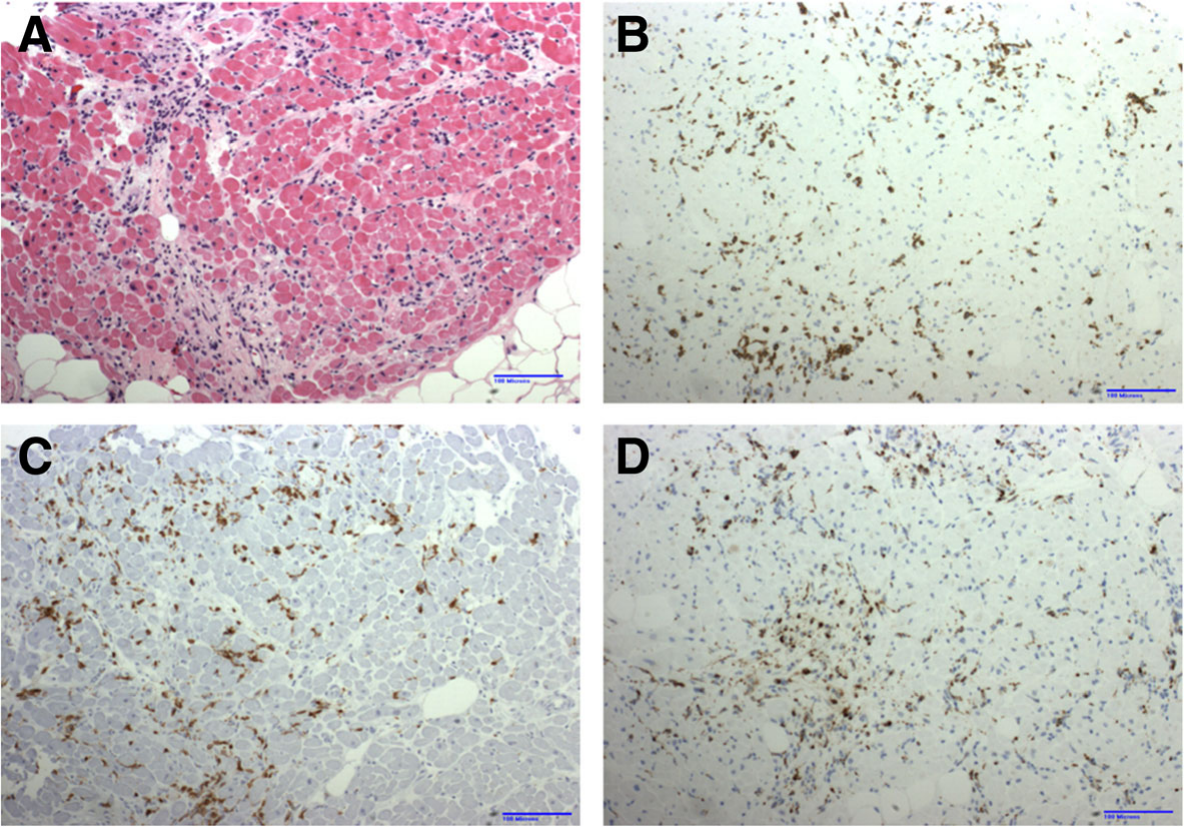
Endomyocardial biopsy revealed. **a** Focal mononuclear inflammatory infiltrate in early collagenized areas. **b** CD3 immunohistochemistry demonstrated abundant T lymphocytes while CD20 (not shown) showed only rare B lymphocytes. **c** CD8 immunohistochemistry showed most T lymphocytes were cytotoxic cells while CD4 staining (not shown) showed positivity in a minority of cells. **d** CD68 revealed also a significant number of macrophages

Two weeks after the first dose of ipi/nivo, she developed nausea unrelieved by ondansetron and proton pump inhibition with normal amylase, lipase and transaminases. Magnetic Resonance Imaging (MRI) of the brain was normal 5 days before CPB and again 4 months later. Additional workup revealed elevated cTnI to 0.19 ng/mL (3× upper limit of normal [ULN]) which more than doubled over the next 4 days to 0.44 ng/mL (7× ULN) (Fig. 1). Creatine kinase–myocardial band (CK-MB), was initially normal but became mildly elevated to 6.3 ng/mL (normal <5 ng/mL). Total creatine kinase was minimally elevated at 335 U/L (normal <190 U/L) indicating no significant myositis. An ECG did not demonstrate conduction delays or ST changes, and echocardiogram showed a left ventricular ejection fraction of ≥ 55% with normal wall motion and no significant valvular abnormalities. Cardiac MRI including T2-weighted spin echo images and late gadolinium enhanced images with an inversion recovery gradient echo sequence was normal with no evidence of focal myocardial fibrosis, scarring or inflammation. She had no active or antecedent symptoms suggestive of viral illness, absolute lymphocyte count was normal, and serology was negative for hepatitis B, hepatitis C, and HIV. Cardiology was consulted and felt that the clinical presentation and workup was not suggestive of ischemia. Checkpoint blockade was discontinued, and intravenous methylprednisolone was initiated at 125 mg daily for presumed immune-mediated myocarditis. Following 3 days of high dose intravenous glucocorticoid, her nausea resolved and cTnI declined to 0.26 ng/mL (4× ULN) with a normal CK-MB level. Steroid therapy was converted to oral prednisone at 1 mg/kg daily and tapered over 1 month. cTnI levels nadired at 0.11 ng/mL (1.8× ULN) but climbed again to 0.78 ng/mL (13× ULN) 2 months after therapeutic dose prednisone was discontinued and 4 months following the only dose of ipi/nivo (Fig. 1). To exclude heterophilic antibodies as a source of false positive cTnI elevation, serum was assayed with and without polyethylene glycol pre-incubation and no antibodies were detected. The patient was negative for rheumatoid factor and renal function remained normal, excluding other potential causes for cTnI elevation. CK-MB, which had been normal for several months, became elevated again at 7.6 ng/mL (1.5× ULN) and total CK was again minimally elevated. The patient was asymptomatic, and repeated ECG, echocardiogram and cardiac MRI were normal. Serum brain natriuretic peptide levels were normal, indicating absence of excessive left ventricular wall stress [6]. To establish a diagnosis, right heart catheterization with endomyocardial biopsy showed areas of early collagen deposition admixed with inflammatory cells (Fig. 2). The inflammation was not seen adjacent to or encroaching on normal cardiac myocytes. Immunohistochemistry (IHC) revealed the great majority of the inflammatory cells were CD3+ T cells and CD68+ macrophages. Furthermore, the majority of the T lymphocytes were CD8+ granzyme B+ cytotoxic cells and a minority CD4+ helper cells. Only rare CD20+ B lymphocytes and CD138+ plasma cells were noted. An exercise SPECT myocardial perfusion study showed no abnormality to explain the clinical or histopathologic findings. Thus, the patient was thought to have CPB-induced smoldering myocarditis persisting for almost 4 months and resulting in myocardial fibrosis with preserved myocardial function.

**Fig. 3:**
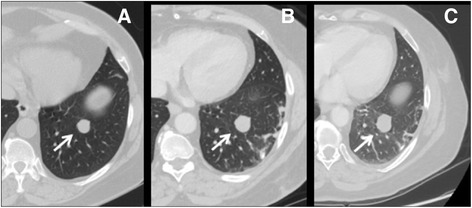
Serial chest CT images showing a representative left lower lobe metastasis over time (arrows). The nodule progressed for 3 months prior to ipi/nivo (image (**a**) to image (**b**)) but remained relatively stable 7 months following immunotherapy (image (**c**)). Three additional pulmonary metastases followed similar clinical courses with no new disease

Computed tomography (CT) scans of the chest, abdomen and pelvis performed 7 months after the initiation of CPB demonstrated stable disease with multiple persistent lung metastases up to 40 mm in diameter (Fig. 3). Due to reluctance to use more potent immunosuppression in the setting of stable metastases, chronic, low-dose prednisone was started at 10 mg daily following the myocardial biopsy. As depicted in Fig. 1, cTnI levels steadily declined during the first 8 weeks of low dose prednisone but subsequently plateaued near 0.3 ng/mL (5× ULN) with persistent mild elevation of CK-MB at 8.4 ng/mL (1.68× ULN) and intermittent atypical chest discomfort at the cardiac apex. Initial measurement of serum anti-striated muscle antibodies was mildly elevated at a titer of 1:120 (Mayo Clinic Laboratory). Intravenous immunoglobulin (IVIg) was administered at 400 mg/kg daily for 2 days beginning 227 days following initiation of CPB. Two weeks later the CK-MB level normalized after being persistently elevated on 8 consecutive determinations over the preceding 4 months and has remained normal on 3 consecutive analyses over 1 month. The rate of decline in cTnI levels also accelerated following intravenous immunoglobulin (Fig. 1) and repeat assay for serum anti-striated muscle antibodies became negative.

## Discussion and conclusions

Although uncommon, myocarditis is an established irAE following CPB, especially combination therapy with ipi/nivo. The immunotherapy literature has focused upon severe cases associated with chest pain, heart block, arrhythmias, heart failure and even sudden death [2, 7]. However, myocarditis of other diverse etiologies may present as incidental detection of elevated serum cTnI with little or no symptoms [6]. Both ECG and cTnI measurements are recommended as initial diagnostic tests for suspected myocarditis and are gradually being adopted as screening tests during early ipi/nivo treatment. However, ECG and even cardiac MRI may be normal in the presence of significant myocarditis [6]. Our patient was diagnosed with CPB-induced myocarditis based upon an acute rise in cTnI beginning 2 weeks after the first dose of ipi/nivo consistent with the reported median onset of clinical myocarditis at 17 days [2, 7], as well as a lack of other causes despite extensive cardiac evaluation. Her presentation was associated with intractable nausea with no known gastrointestinal etiology. High dose glucocorticoid therapy led to rapid resolution of nausea and a four-fold decrease in cTnI over 4 days. Serum cTnI spiked again following a steroid taper with endomyocardial biopsy revealing collagen fibrosis and lymphocytic inflammation consistent with chronic smoldering myocarditis which responded gradually to chronic prednisone at 10 mg daily.

Since subclinical myocarditis following immune CPB has not been previously described, the natural history of this entity is unclear but may parallel viral myocarditis. Most chronic viral myocarditis is lymphocytic and autoimmune where initial viral infection of the myocardium leads to recognition of self-antigens with persistent anti-cardiac antibodies and inflammation despite viral clearance based upon endomyocardial biopsy [8]. Myocardial fibrosis is frequently co-localized with inflammation and contributes to chronic dilated cardiomyopathy and ventricular arrhythmias [9]. Early diagnosis and treatment with prednisone or cyclosporine improves long term outcomes [8]. Myocardial fibrosis has also been reported in three patients with cardiotoxicity associated with CPB [4]. Another possibility is that the patient would have developed fulminant myocarditis, but this outcome was prevented with early corticosteroid use. Our case together with several recent reports strongly suggests the need for additional research involving large patient cohorts with serial ECGs and cTnI measurements especially during the first 2 months of immune CPB to better characterized the incidence of subclinical myocarditis, its natural history and response to treatment to potentially reduce long-term sequelae of myocardial fibrosis and dilated cardiomyopathy or reduce progression to fulminant myocarditis [2, 4, 7, 10]. For patients receiving immunotherapy who experience unexplained troponinemia, endomyocardial biopsy should be considered to look for pathological evidence of myocarditis according to the Dallas criteria [11, 12].

Under physiologic conditions, CTLA-4 and PD-1 are known to protect the heart from immune-mediated damage. Specifically, CTLA-4 knockout mice develop rapidly fatal autoimmune myocarditis mediated by CD8+ T cells while PD-1 knockout mice survive longer but also develop spontaneous myocarditis and dilated cardiomyopathy [13]. T cells and macrophages infiltrate the myocardium, and cardiac specific auto-antibodies are thought to play a major pathogenic role in some genetic backgrounds [14, 15]. In models of T-cell-mediated myocarditis, PD-L1 up-regulation in cardiac myocytes appears to be a cytokine-induced cardio-protective mechanism that is abrogated by immune CPB [2, 16, 17].

Once a diagnosis of immune-mediated myocarditis is strongly suspected, immune CPB should be discontinued and immunosuppression promptly initiated based upon the severity of the presentation [7]. In clinically ambiguous situations, endomyocardial biopsy should be considered before CPB is permanently discontinued to avoid unnecessary withdrawal of potentially effect therapy for the underlying malignancy. High dose glucocorticoids are recommended for severe cases, but our experience indicates chronic, low-dose prednisone may be sufficient for subclinical presentations. Our patient demonstrated evidence of a humoral immune component with elevation of serum anti-striated muscle antibodies and rapid normalization of CK-MB levels following IVIg. Although infliximab is commonly used for steroid-refractory colitis or pneumonitis following CPB, it has been associated with heart failure [18]. Anti-thymocyte globulin or tacrolimus may be considered in refractory cases given their success in treating cardiac allograft rejection [7].

This case demonstrates that subclinical, smoldering myocarditis may occur following immune checkpoint blockade and respond to corticosteroid therapy. It is unclear whether this case represents an early presentation of fulminant myocarditis that was forestalled with prompt immunosuppression, or a more subacute process with distinct pathophysiology. Regardless, this experience supports early monitoring for myocarditis in large, prospective cohorts of patients receiving combination CPB, which is becoming more widely used to treat a broad array of malignancies in patients achieving long term survival.

## Abbreviations

CD#: Cluster of differentiation #
CK: Creatine kinase
CK-MB: Creatine kinase-myocardial band
CPB: Checkpoint blockade
CTLA-4: Cytotoxic T lymphocyte associated protein 4
cTnI: Cardiac troponin I
ECG: Electrocardiogram
HIV: Human immunodeficiency virus
IHC: Immunohistochemistry
irAE: Immune-related adverse event
IVIg: Intravenous immunoglobulin
MRI: Magnetic resonance imaging
PD-1: Programmed cell death protein 1
PD-L1: Programmed cell death protein ligand 1
SPECT: Single-photon emission computer tomography
ULN: Upper limit of normal

## References

[1] Harris SJ, Brown J, Lopez J (2016). Immuno-oncology combinations: raising the tail of the survival curve. Cancer Biol Med.

[2] Johnson DB, Balko JM, Compton ML (2016). Fulminant Myocarditis with combination immune checkpoint blockade. N Engl J Med.

[3] Laubli H, Balmelli C, Bossard M (2015). Acute heart failure due to autoimmune myocarditis under pembrolizumab treatment for metastatic melanoma. J Immunother Cancer.

[4] Heinzerling L, Ott PA, Hodi S (2016). Cardiotoxicity associated with CTLA4 and PD1 blocking immunotherapy. J Immunother Cancer.

[5] Makaryus AN, Makaryus MN, Hassid B (2007). Falsely elevated cardiac troponin I levels. Clin Cardiol.

[6] Gaborit F, Bosselmann H, Tonder N (2015). Association between left ventricular global longitudinal strain and natriuretic peptides in outpatients with chronic systolic heart failure. BMC Cardiovasc Disord.

[7] Wang DY, Okoye GD, Neilan TG (2017). Cardiovascular toxicities associated with cancer Immunotherapies. Curr Cardiol Rep.

[8] Rose NR (2016). Viral myocarditis. Curr Opin Rheumatol.

[9] Disertori M, Mase M, Ravelli F (2017). Myocardial fibrosis predicts ventricular tachyarrhythmias. Trends Cardiovasc Med.

[10] Voskens CJ, Goldinger SM, Loquai C (2013). The price of tumor control: an analysis of rare side effects of anti-CTLA-4 therapy in metastatic melanoma from the ipilimumab network. PLoS One.

[11] Cooper LT, Baughman KL, Feldman AM (2007). The role of endomyocardial biopsy in the management of cardiovascular disease: a scientific statement from the American Heart Association, the American College of Cardiology, and the European Society of Cardiology. Endorsed by the Heart Failure Society of America and the heart failure Association of the European Society of cardiology. J Am Coll Cardiol.

[12] Aretz HT (1987). Myocarditis: the Dallas criteria. Hum Pathol.

[13] Love VA, Grabie N, Duramad P (2007). CTLA-4 ablation and interleukin-12 driven differentiation synergistically augment cardiac pathogenicity of cytotoxic T lymphocytes. Circ Res.

[14] Lucas JA, Menke J, Rabacal WA (2008). Programmed death ligand 1 regulates a critical checkpoint for autoimmune myocarditis and pneumonitis in MRL mice. J Immunol.

[15] Okazaki T, Tanaka Y, Nishio R (2003). Autoantibodies against cardiac troponin I are responsible for dilated cardiomyopathy in PD-1-deficient mice. Nat Med.

[16] Tarrio ML, Grabie N, Bu DX (2012). PD-1 protects against inflammation and myocyte damage in T cell-mediated myocarditis. J Immunol.

[17] Grabie N, Gotsman I, DaCosta R (2007). Endothelial programmed death-1 ligand 1 (PD-L1) regulates CD8+ T-cell mediated injury in the heart. Circulation.

[18] Kwon HJ, Cote TR, Cuffe MS (2003). Case reports of heart failure after therapy with a tumor necrosis factor antagonist. Ann Intern Med.

